# Evaluation of clindamycin use in bone and joint infections: is monotherapy a safe option? A monocentric observational study 2014–19

**DOI:** 10.1093/jacamr/dlaf164

**Published:** 2025-09-23

**Authors:** Simon Jamard, Marie-Frédérique Lartigue, Louis-Romée Le Nail, Vianney Tuloup, Marion Lacasse, Adrien Lemaignen, CRIOGO local group, Laura Chaufour, Geoffroy Dubois De Montmarin, Marion Lacasse, Marie-Frédérique Lartigue, Adrien Lemaignen, Louis-Romée Le Nail, Denis Mulleman, Vianney Tuloup

**Affiliations:** Service de Maladies Infectieuses et Tropicales, Hôpital Bretonneau, CHRU de Tours, Tours 37044, France; UMR1282, Infectiologie et Santé Publique, BRMF, Université de Tours, INRAE, Tours, France; UMR1282, Infectiologie et Santé Publique, BRMF, Université de Tours, INRAE, Tours, France; Service de Bactériologie et Hygiène Hospitalière, Hôpital Trousseau, CHRU de Tours, Tours 37044, France; Service de Chirurgie Orthopédique, Hôpital Trousseau, CHRU de Tours, Tours 37044, France; Pharmacie à Usage Intérieur, Hôpital Trousseau, CHRU de Tours, Tours 37044, France; Service de Maladies Infectieuses et Tropicales, Hôpital Bretonneau, CHRU de Tours, Tours 37044, France; Service de Maladies Infectieuses et Tropicales, Hôpital Bretonneau, CHRU de Tours, Tours 37044, France; EA 7505, Education, Ethique, Santé, Université de Tours, Tours, France

## Abstract

**Background:**

Selecting an optimal antibiotic regimen for bone and joint infections (BJIs) is challenging due to limited high-quality evidence. Although clindamycin is widely used as an alternative treatment for susceptible microorganisms in combination therapy, its use as monotherapy is increasing. This study aimed to evaluate the efficacy and safety of clindamycin monotherapy for BJI treatment.

**Methods:**

A monocentric observational study was conducted using data from the Reference Centre for complex BJI at our tertiary university hospital between 2014 and 2019. All adult patients with microbiologically confirmed BJI receiving clindamycin after a multidisciplinary meeting were included. Patients infected with clindamycin non-susceptible strains were excluded. Treatment failure was defined as relapse, treatment change or death from any cause within 1 year. Associations between monotherapy and treatment failure were assessed using multivariate logistic regression and inverse probability of treatment weighting (IPTW) analysis to adjust for the propensity to receive monotherapy.

**Results:**

A total of 137 patients were included, of whom 88 received clindamycin monotherapy. Overall, 41/137 treatment failures were observed (16/88 in the monotherapy group, 25/49 in the combination group). Monotherapy was associated with fewer failures in both multivariate (OR = 0.18; 95% CI, 0.07–0.46; *P* < 0.001) and IPTW-adjusted models (OR = 0.36; 95% CI, 0.17–0.76; *P* = 0.008). Patients treated with monotherapy presented with milder infections, less fever and lower Charlson comorbidity scores, with significantly lower baseline C-reactive protein levels (102.6 versus 65.7 mg/L; *P* = 0.006). Fewer adverse events were reported in the monotherapy group (4/88 versus 8/49, *P* = 0.04).

**Conclusions:**

Clindamycin monotherapy appears to be a reliable and safe therapeutic option for selected patients with less severe BJI.

## Introduction

Bone and joint infections (BJIs) are a heterogeneous and complex group of conditions with an increasing incidence, currently estimated at 70 cases per 100 000 inhabitants annually in France.^1–3^ Their management requires a multidisciplinary approach combining medical and surgical interventions, tailored according to factors such as the duration of infection, anatomical site, chronicity and the presence of foreign material.^4^ Despite advances in treatment, BJIs remain associated with a therapeutic failure rate of approximately 20%, a mortality rate of 5%, and a significant impact on quality of life in up to 40% of survivors.^2^ Consequently, BJIs represent a major public health concern with substantial healthcare costs. To address these challenges, France established a national network of reference centres for complex BJI management (CRIOAc: Centres de Référence des Infections Ostéo-Articulaires complexes) in 2008. These centres provide specialized care through multidisciplinary meetings (MMs), and contribute to research and education in the field.^5^

Antibiotic therapy for BJIs is guided by microbiological findings, with *Staphylococcus* spp. accounting for approximately 50.6% of cases.^6^ First-line agents typically include β-lactams, fluoroquinolones and rifampicin, often used in combination. Clindamycin is recommended as a second-line agent or as an alternative in cases of allergy, particularly for infections caused by *Staphylococcus*, *Streptococcus* and anaerobic bacteria, regardless of the BJI type.^7–9^

Clindamycin, a lincosamide antibiotic, exerts its antibacterial effect by binding to the 50S ribosomal subunit, inhibiting protein synthesis in aerobic Gram-positive and anaerobic organisms.^10^ Its favourable pharmacokinetic profile—including 90% oral bioavailability, excellent bone penetration^11,12^ and effective intracellular activity against intraosteoblastic bacterial reservoirs^13^—makes it a valuable option in BJI treatment.^14–16^ These properties have led to its increasing use, with clindamycin prescribed in 10.7% of BJIs managed within the CRIOAc,^6^ often in combination regimens.

However, concerns have been raised regarding its use in combination with rifampicin, as recent studies have demonstrated a significant reduction in clindamycin serum concentrations due to hepatic enzyme induction by rifampicin.^17,18^ Despite this pharmacokinetic interaction, clinical efficacy seems to be preserved,^19^ and the combination remains endorsed by French guidelines.^7–9^ Concurrently, antimicrobial stewardship efforts advocate for minimizing unnecessary antibiotic combinations to reduce resistance development.

In real-life practice, combination therapy may not always be feasible due to drug interactions or patient comorbidities. Nevertheless, data on the efficacy and safety of clindamycin monotherapy in BJI remain limited, with existing evidence largely derived from older studies^20^ or subgroup analysis^14^ that suggest no increased risk of treatment failure.

The present study aimed to describe the use of clindamycin in our BJI cohort, and to evaluate the efficacy of clindamycin monotherapy compared with combination therapy. Secondary objectives included assessing treatment tolerance and identifying risk factors associated with therapeutic failure.

## Methods

### Study design and population

This monocentric observational study was conducted using the cohort of the Referral Centre for BJI (CRIOAc) of Tours University Hospital, France, between 2014 and 2019. Eligible participants were adults (≥18 years) with a confirmed diagnosis of BJI, as validated during MMs. Diagnosis required a compatible clinical and radiological presentation, along with positive microbiological findings—defined as either a positive blood culture or joint/surgical sample with at least two concordant isolates in cases of potential contaminants. Inclusion was limited to patients for whom an antibiotic regimen including clindamycin was recommended during MMs.

Exclusion criteria comprised: (i) infections involving at least one bacterial strain resistant to clindamycin; (ii) patients who did not receive any dose of clindamycin; (iii) patients followed up in another centre without accessible outcome data; and (iv) patients who explicitly declined participation.

### Data collection

Eligible patients were identified from the local CRIOAc database, which records all cases discussed during the weekly MM of the Referral Centre. This database includes demographic characteristics, medical history, clinical and microbiological data, and details of antimicrobial and surgical management. Follow-up data, clinical outcome and adverse events were not included in the CRIOAc database and were completed from electronic patient records.

### Outcomes

The primary outcome was the occurrence of treatment failure at 1 year follow-up. This composite outcome was defined as: (i) modification of antimicrobial therapy, (ii) requirement for additional surgery due to persistent or recurrent infection at the same site (based on clinical, microbiological or radiological criteria), or (iii) death for any cause.

Secondary outcome was the occurrence of adverse events, including gastrointestinal intolerance (nausea, vomiting, diarrhoea), *Clostridioides difficile* colitis, hypersensitivity reaction, drug-induced hepatitis or renal impairment.

### Statistical analysis

All statistical analysis and tables were generated using R version 4.2.3 (R Foundation for Statistical Computing; https://www.R-project.org/) and RStudio (v2023.3.0.0). Figures were generated using GraphPad Prism (v8.0.2). Statistical significance was established at α = 0.05. Continuous variables were compared using Student’s *t*-test or the Wilcoxon rank-sum test, as appropriate. Categorical variables were compared using the χ^2^ test or Fisher’s exact test. Logistic regression was used to identify factors associated with treatment failure and adverse events. Variables with a *P* value <0.30 in univariate analysis were included in multivariate models,^21^ after assessing for potential interactions. Model selection was guided by minimization of the Akaike information criterion.^22^

To address potential confounding, an inverse propensity of treatment weighting (IPTW) approach was employed.^23^ The propensity score—defined as the probability of receiving clindamycin monotherapy based on baseline covariates—was estimated using variables listed in Table S1 (available as Supplementary data at *JAC-AMR* Online). To check the ability of IPTW to obtain well-balanced arms and thereby correct for confounding, standardized differences between arms before and after weighting were computed. A standardized difference <10% was considered as indicating successful balancing and was used as a target in the construction of the propensity score, acknowledging that there was no guarantee it would be achieved.

## Results

### Study population

From 2014 to 2019, 2131 patients were presented at MMs of our CRIOAc for a suspected BJI. Among them, 252 patients received a recommendation for clindamycin-based therapy, of whom 137 met the inclusion criteria. Exclusions included: 63 patients with infections caused by at least one clindamycin-resistant organism, 22 with no microbiological identification, 27 who did not receive clindamycin despite MM recommendation, and 3 patients lost to follow-up due to care provided at another centre. Clindamycin was administered orally at a dose of 600 mg three times a day (or four times a day if BMI > 30 kg/m^2^), as an oral step-down after an initial 7–10 day course of IV empirical therapy with a comination of piperacillin/tazobactam and linezolid. Of the 137 included patients, 88 received clindamycin monotherapy (monotherapy group) and 49 in combination with at least one other antibiotic (combination group) (Figure S1).

### Patient characteristics

Patients were predominantly male (99/137, 72.3%), with a median age of 66 years (Table 1). They had multiple comorbidities, with a median adjusted Charlson score of 6 (IQR: 5). Nearly half of the patients were diabetic, 57/137 (41.6%) were active smokers, and 48/137 (35%) reported excessive alcohol consumption. Chronic alcoholic intoxication and severe liver disease were significantly more prevalent in the combination group (respectively, 23/49 versus 25/88, *P* = 0.04; and 9/49 versus 4/88, *P* = 0.02).

**Table 1.** Baseline characteristics of patients and BJI episode^a^

	In combination (*n* = 49)	Monotherapy (*n* = 88)	Total (*n* = 137)	*P* value
Demographics				
Sex (male)	36 (73.5)	63 (71.6)	99 (72.3)	0.97
Age, median (IQR), y	66.00 (22.00)	65.50 (21.25)	66.0 (21.0)	0.36
Medical history				
Charlson score, median (IQR)	6.00 (6.00)	5.00 (5.00)	6.00 (5.00)	0.08
Chronic kidney failure, eGFR < 60 mL/min	14 (28.6)	21 (23.9)	35 (25.5)	0.68
Chronic heart failure	12 (24.5)	28 (31.8)	40 (29.2)	0.48
Ischaemic heart disease	6 (12.2)	11 (12.5)	17 (12.4)	>0.99
Arterial occlusive diseases	6 (12.2)	16 (18.2)	22 (16.1)	0.51
Stroke	6 (12.2)	9 (10.2)	15 (10.9)	0.94
Hemiplegia/paraplegia/quadriplegia	3 (6.1)	2 (2.3)	5 (3.6)	0.49
Dementia	5 (10.2)	5 (5.7)	10 (7.3)	0.53
Obesity (BMI > 30 kg/m^2^)	15 (30.6)	24 (27.3)	39 (28.5)	0.83
Malnutrition	2 (4.1)	5 (5.7)	7 (4.14)	0.99
Chronic respiratory pathology	13 (26.5)	14 (15.9)	27 (19.7)	0.20
Cirrhosis stage B or more	9 (18.4)	4 (4.5)	13 (9.5)	0.02*
Diabetes mellitus	23 (46.9)	41 (46.6)	64 (46.7)	>0.99
Malignant neoplasm	9 (18.4)	19 (21.6)	28 (20.4)	0.82
Haematological malignancy	1 (2)	3 (3.4)	4 (2.9)	>0.99
Immunodeficiency	10 (20.4)	17 (19.3)	27 (19.7)	>0.99
Uncontrolled HIV infection or HIV infection with CD4 cell counts <200/µL	2 (4.1)	1 (1.1)	3 (2.2)	0.60
Inflammatory rheumatism	5 (10.2)	11 (12.5)	16 (11.7)	0.90
Active smoking habit	25 (51)	32 (36.4)	57 (41.6)	0.14
Chronic alcoholic intoxication	23 (46.9)	25 (28.4)	48 (35)	0.04*
History of *Clostridioides* colitis	0 (0)	1 (1.1)	1 (0.7)	>0.99
Characteristics of infection				
Number of different sites				0.37
Mono- (1)	47 (96)	87 (98.9)	134 (97.8)	
Oligo- (2–3)	1 (2)	1 (1.1)	2 (1.5)	
Poly- (>3)	1 (2)	0 (0)	1 (0.7)	
Site of infection				0.91
Axial	2 (4.1)	3 (3.4)	5 (3.7)	
Lower limbs	35 (71.4)	60 (68.2)	95 (69.3)	
Upper limbs	11 (22.5)	24 (37.3)	35 (25.5)	
Multiple sites	1 (2)	1 (1.1)	2 (1.5)	
Diabetic foot–related infection^b^	2 (4.1)	7 (7.9)	9 (6.6)	0.61
Arthritis^b^	11 (22.5)	11 (12.5)	22 (16.1)	0.20
Osteitis^b^	11 (22.5)	19 (21.6)	30 (21.9)	>0.99
Device-related infection^b^	27 (55.1)	57 (64.8)	84 (61.3)	0.35
Of which: prosthetic joint infection	13 (26.5)	32 (36.4)	45 (32.8)	0.33
Type of prosthesis				0.90
Knee	3 (6.1)	5 (5.7)	8 (5.8)	
Hip	9 (18.4)	19 (21.6)	28 (20.4)	
Chronic infection	38 (77.6)	63 (71.6)	101 (73.7)	0.58
First episode of infection	28 (57.1)	54 (61.4)	82 (59.8)	0.76
C-reactive protein, median (IQR), mg/L	102.6 (104)	65.7 (80.8)	68.5 (100)	0.006*
Fever	25 (51)	29 (32.9)	54 (39.4)	0.06
Microbiological identification				
Polymicrobial infection	12 (24.5)	27 (30.7)	39 (28.5)	0.57
*Staphylococcus aureus*	29 (59.2)	52 (59.1)	81 (59.1)	>0.99
Coagulase-negative staphylococci	16 (32.6)	24 (27.3)	40 (29.2)	0.64
Group A streptococci	2 (4.1)	0 (0.00)	2 (1.5)	0.24
Group B streptococci	1 (1)	2 (2.3)	3 (2.2)	>0.99
Other streptococci	2 (4.18)	9 (10.2)	11 (8)	0.35
*Cutibacterium* sp.	5 (10.2)	15 (17.1)	20 (14.6)	0.40
Other anaerobes	4 (8.2)	2 (2.3)	6 (4.4)	0.24
Other bacteria	2 (4.1)	3 (3.4)	5 (3.7)	>0.99
Treatment and outcome				
Surgery	45 (91.8)	82 (93.2)	127 (92.7)	>0.99
Duration of treatment, median (IQR), d	42 (3)	42 (3)	42 (3)	0.25
Failure	25 (51)	16 (18.2)	41 (29.9)	<0.001*
Clinical failure	17 (34.7)	14 (15.9)	31 (22.6)	0.02*
Microbiological failure	9 (18.4)	6 (6.8)	15 (10.9)	0.07
Radiological failure	7 (14.3)	4 (4.5)	11 (8)	0.09
Adverse events	8 (16.3)	4 (4.5)	12 (8.8)	0.04*
Gastrointestinal event	3 (6.1)	1 (1.1)	4 (2.9)	0.26
Of which: *Clostridioides difficile* colitis	2 (4.18)	0 (0)	2 (1.5)	0.24
Allergy	3 (6.1)	2 (2.3)	5 (3.6)	0.50
Acute renal failure	2 (4.1)	1 (1.1)	3 (2.2)	0.60

eGFR, estimated glomerular filtration rate.

^a^All values are *n* (%) except where noted.

^b^Patients with multisite infections were counted more than once for each infected site.

^*^Statistically significant with *P* value <0.05.

Most infections (134/137) were localized to a single site, primarily the lower limbs. Device-related infections were the most frequent (84/137), with prosthetic joint infections (PJIs) accounting for over half (45/84). Diabetic foot infections were relatively rare (9/137). The majority of cases were chronic infections (101/137, 73.7%) defined as lasting more than 1 month, and this infection was the first episode for 82 cases. At baseline, C-reactive protein (CRP) levels were significantly higher in the combination group (median: 102.6 mg/L versus 65.7 mg/L, *P* = 0.006) and fever was more frequent [25/49 (51%) versus 29/88 (32.9%)].

*Staphylococcus aureus* was the most commonly isolated pathogen, in 81/137 (59.1%), followed by CoNS in 40/137 (29.2%) and *Cutibacterium* sp. in 20/137 (14.6%). Polymicrobial infections were observed in 39/137 cases. All strains were susceptible to clindamycin. Notably, CoNS exhibited resistance to erythromycin, one by an efflux pump and the other by an inducible MLS_b_ phenotype. Both erythromycin-resistant strains were treated with clindamycin monotherapy.

**Figure 1.**
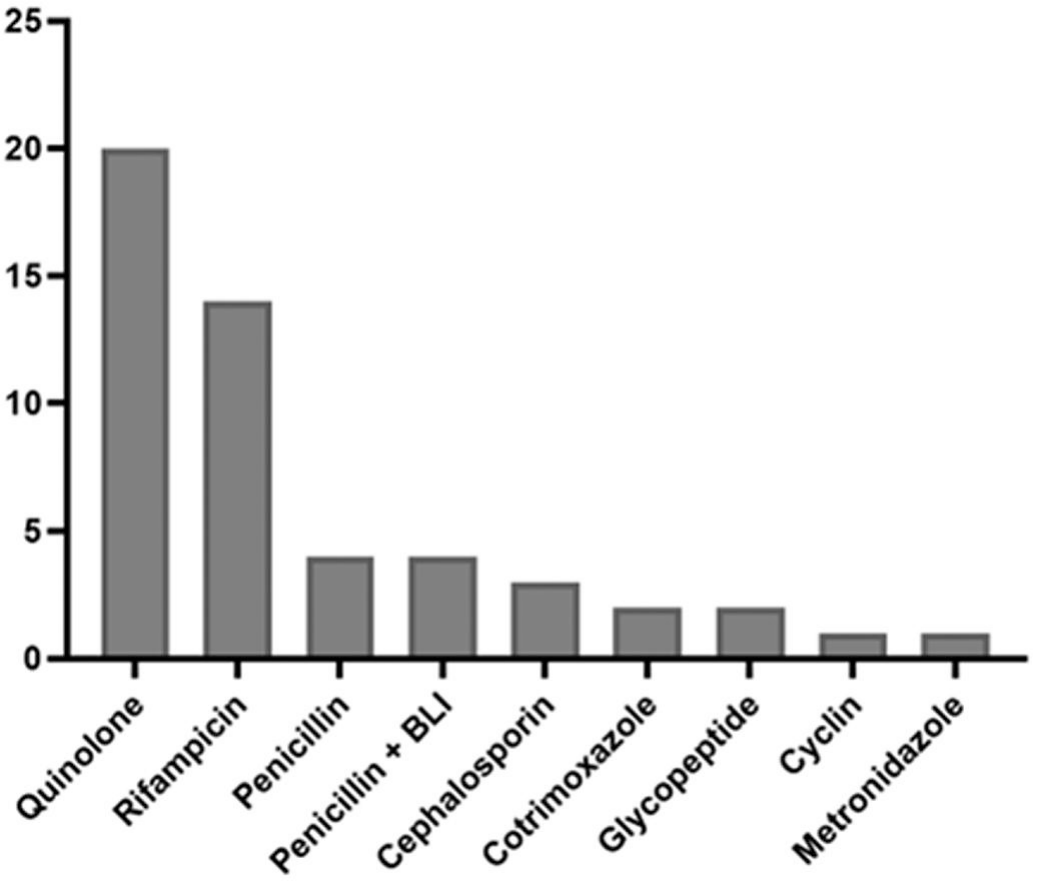
Treatment associated with clindamycin for patients treated in combination regimen. Forty-seven patients were treated with a combination of two antibiotics. Two patients were treated with a combination of three antibiotics: clindamycin/fluoroquinolone/rifampicin and clindamycin/penicillin/rifampicin. BLI, β-lactamase inhibitor.

Surgical intervention was performed in 127/137 (92.7%) patients, primarily debridement in 70/127 (55%), with implant retention if an infected device was involved. All patients received clindamycin-based therapy. In the combination group, the most frequently co-administered antibiotics were fluoroquinolones (20/49) and rifampicin (14/49) (Figure 1). Forty-seven of 49 patients received dual therapy, whereas 2 received triple therapy: clindamycin/ fluoroquinolone/ rifampicin and clindamycin/ penicillin/ rifampicin. The median duration of treatment was 42 days.

### Treatment outcome and risk factors for failure

After a mean follow-up of 438 days (SD: 415), 41 patients experienced treatment failure: 31 clinical, 15 microbiological and 11 radiological failures. Treatment failure was significantly more frequent in the combination group compared with the monotherapy group: 17/49 (51%) versus 16/88 (18.2%); *P* < 0.001.

Univariate analysis identified arterial occlusive disease (OR = 2.83; 95% CI, 1.11–7.29; *P* = 0.03), chronic respiratory pathology (OR = 2.72; 95% CI, 1.13–6.53; *P* = 0.02) and cirrhosis (OR = 4.41; 95% CI, 1.37–15.51; *P* = 0.01) as significantly associated with treatment failure (Table 2). These associations were not retained in multivariate and adjusted analysis. Malnutrition was consistently associated with treatment failure across univariate (OR = 6.53; 95% CI, 1.34–47.03; *P* = 0.03), multivariate (OR = 11.83; 95% CI, 1.61–118.87; *P* = 0.02) and adjusted models (OR = 11.83; 95% CI, 1.74–112.31; *P* = 0.02).

**Table 2.** Analysis of risk factors for treatment failure^a^

	Success (*n* = 96)^b^	Failure (*n* = 41)^b^	Univariate	Multivariate	Multivariate adjusted on AIC	IPTW
			OR (95% CI; *P*)	OR (95% CI; *P*)	OR (95% CI; *P*)	OR (95% CI; *P*)
Arterial occlusive diseases	11 (11.5)	11 (26.8)	2.83 (1.11–7.29; *P* = 0.03)	1.91 (0.46–7.87; *P* = 0.34)	2.81 (0.89–9.07; *P* = 0.08)	—
Malnutrition	2 (2.1)	5 (12.2)	6.53 (1.34–47.03; *P* = 0.03)	11.83 (1.61–118.87; *P* = 0.02)	11.83 (1.74–112.31; *P* = 0.02)	—
Chronic respiratory pathology	14 (14.6)	13 (31.7)	2.72 (1.13–6.53; *P* = 0.02)	1.35 (0.40–4.34; *P* = 0.61)	—	—
Cirrhosis stage B or more	5 (5.2)	8 (19.5)	4.41 (1.37–15.51; *P* = 0.01)	2.05 (0.39–11.73; *P* = 0.4)	3.17 (0.80–13.64; *P* = 0.1)	—
Chronic alcoholic intoxication	29 (30.2)	19 (46.3)	2.00 (0.94–4.25; *P* = 0.07)	1.46 (0.52–4.10; *P* = 0.46)	—	—
Type of prothesis						
Knee	3 (3.1)	5 (12.2)	3.94 (0.91–20.23; *P* = 0.07)	3.93 (0.52–34.28; *P* = 0.19)	—	—
C-reactive protein, median (IQR), mg/L	64.85 (82.85)	102.6 (124.0)	1.01 (1.00–1.01; *P* = 0.008)	1.00 (0.99–1.01; *P* = 0.87)	—	—
Fever	30 (31.2)	24 (58.5)	3.11 (1.47–6.71; *P* = 0.003)	2.88 (1.00–8.73; *P* = 0.05)	3.26 (1.30–8.56; *P* = 0.01)	—
Monotherapy	72 (75.0)	16 (39.0)	0.21 (0.10–0.46; *P* < 0.001)	0.19 (0.06–0.51; *P* = 0.001)	0.18 (0.07–0.46; *P* < 0.001)	0.36 (0.17–0.76; *P* = 0.008)
Duration of treatment, median (IQR), d	42.0 (3.0)	42.0 (3.0)	1.00 (0.99–1.02; *P* = 0.57)	—	—	—

^a^Logistic regression was in univariate and multivariate models, based on variables with *P* < 0.3 in univariate analysis. The multivariate model was completed with an adjusted multivariate analysis based on the selection of the best fitted model by maximizing the Akaike information criterion. An IPTW was also performed to assess the bias of treatment selection. The complete logistic regression is given in Table S3.

^b^Values are *n* (%) except where noted otherwise.

Among baseline infection characteristics, CRP levels (OR = 1.01; 95% CI, 1.00–1.01; *P* = 0.008), and fever (OR = 3.11; 95% CI, 1.47–6.71; *P* = 0.003) were significantly associated with treatment failure, though only fever remained significant in multivariate and adjusted models.

Clindamycin monotherapy was strongly associated with a favourable outcome, with consistent results across multivariate (OR = 0.19; 95% CI, 0.06–0.51; *P* = 0.001) and adjusted (OR = 0.18; 95% CI, 0.07–0.46; *P* < 0.001) models. To further explore this association, an IPTW analysis was conducted to assess the parameters and possible bias responsible for a patient to be administered clindamycin in monotherapy instead of a combination regimen. The weighted population is described in Table S2. Monotherapy remained significantly associated with favourable outcomes in the IPTW model (OR = 0.36; 95% CI, 0.17–0.76; *P* = 0.008).

A subgroup analysis of common combination regimens (*n* > 3) revealed no increased risk of failure with clindamycin/rifampicin, whereas clindamycin/fluoroquinolone was associated with higher failure rates (Figure S2). A second subgroup analysis focused on the 31 patients with PJIs treated with debridment, antibiotic and implant retention (DAIR). Treatment failure occurred in 4/20 (33%) of monotherapy patients and 8/11 (67%) of combination therapy patients (*P* = 0.012).

### Adverse events

Adverse events were reported for 12 patients, including hypersensitivity reaction (*n* = 5), gastrointestinal events (*n* = 4)—including two cases of colitis due to *Clostridioides difficile* infection—and three acute renal failures. Adverse events were significantly more frequent in the combination group [8/45 (16.3%) versus 4/88 (4.5%), *P* = 0.04]. Monotherapy was associated with a reduced risk of adverse events in univariate analysis (OR = 0.24; 95% CI, 0.06–0.82; *P* = 0.03) (Table 3), though this association was not confirmed in multivariate or IPTW models.

**Table 3.** Analysis of risk factors for adverse events^a^

	No adverse event (*n* = 125)^b^	Adverse event (*n* = 12)^b^	Univariate	Multivariate	Multivariate adjusted on AIC	IPTW
			OR (95% CI; *P*)	OR (95% CI; *P*)	OR (95% CI; *P*)	OR (95% CI; *P*)
Age, median (IQR), y	64 (21)	73 (23)	1.04 (1.00–1.10; *P* = 0.05)	1.01 (0.97–1.07; *P* = 0.58)	—	—
Chronic kidney failure, eGFR < 60 mL/min	30 (24.0)	5 (41.7)	2.26 (0.63–7.62; *P* = 0.19)	2.28 (0.49–10.52; *P* = 0.28)	—	—
Chronic respiratory pathology	21 (16.8)	6 (50.0)	4.95 (1.42–17.33; *P* = 0.01)	2.49 (0.50–11.98; *P* = 0.25)	3.87 (1.02–14.71; *P* = 0.04)	—
Cirrhosis stage B or more	12 (9.6)	1 (8.3)	0.86 (0.04–5.02; *P* = 0.89)	—	—	—
Inflammatory rheumatism	13 (10.4)	3 (25.0)	2.87 (0.58–11.12; *P* = 0.14)	1.59 (0.22–9.44; *P* = 0.61)	—	—
Device-related infection	74 (59.2)	10 (83.3)	3.45 (0.86–23.03; *P* = 0.12)	3.75 (0.78–28.77; *P* = 0.13)	4.42 (1.01–31.57; *P* = 0.08)	—
First episode of infection	73 (58.4)	9 (75.0)	2.14 (0.60–9.98, *P* = 0.27)	1.18 (0.25–6.42; *P* = 0.83)	—	—
C-reactive protein, median (IQR), mg/L	68.1 (88.0)	122.0 (123.25)	1.00 (1.00–1.01; *P* = 0.15)	1.00 (0.99–1.01; *P* = 0.8)	—	—
Fever	46 (36.8)	8 (66.7)	3.43 (1.02–13.45; *P* = 0.05)	2.26 (0.42–12.66; *P* = 0.34)	—	—
Polymicrobial infection	38 (30.4)	1 (8.3)	0.21 (0.01–1.13; *P* = 0.14)	0.31 (0.02–1.93; *P* = 0.29)	0.24 (0.01–1.44; *P* = 0.19)	—
Monotherapy	84 (67.2)	4 (33.3)	0.24 (0.06–0.82; *P* = 0.03)	0.29 (0.06–1.20; *P* = 0.09)	0.26 (0.06–0.97; *P* = 0.053)	0.24 (0.04–1.54; *P* = 0.12)
Duration of treatment, median (IQR), d	42 (3)	42 (0.75)	1.00 (0.97–1.02; *P* = 0.73)	—	—	—

^a^Logistic regression was in univariate and multivariate models, based on variables with *P* < 0.3 in univariate analysis. The multivariate model was completed with an adjusted multivariate analysis based on the selection of the best fitted model by maximizing the Akaike information criterion. An IPTW was also performed to assess the bias of treatment selection. The complete univariate logistic regression is given in Table S4.

^b^Values are *n* (%) except where noted otherwise.

## Discussion

Clindamycin is an antibiotic widely used in the management of BJI, particularly in combination regimens. To our knowledge, this study is the first to specifically compare clindamycin monotherapy with combination therapy with another antibiotic for the treatment of BJI, demonstrating its reliable safety profile, including for device-associated infections. In addition, a monotherapy regimen appears to be better tolerated than the combination regimen with fewer adverse events.

Despite an overall failure rate of approximately 30%, consistent with previous reports,^1^ failure was markedly more frequent in the combination group, exceeding 50%. This discrepancy may reflect the greater clinical complexity of patients in the combination group. Compared with prior CRIOAc network data,^6^ our cohort exhibited a higher prevalence of comorbidities known to increase the risk of treatment failure, including chronic kidney disease (25.5% versus 8.3%), diabetes (46.7% versus 18.3%), tobacco use (41.6% versus 9.7%) and immunodeficiency (19.7% versus 5.1%). These factors may have influenced the decision to prescribe clindamycin, particularly in patients for whom first-line agents were contraindicated due to drug interactions or organ dysfunction.

As with all retrospective studies, confounding by indication remains a concern. Patients treated with a combination regimen exhibited slightly higher rates of comorbidities compared with those receiving monotherapy, especially for chronic liver disease and chronic alcohol intoxication. Furthermore, the combination group faced more severe and complex infections, as evidenced by significantly higher CRP levels, a trend of higher incidence of fever, and lower occurrence of upper limb infections. To address this, we conducted an IPTW analysis, which confirmed the robustness of our findings: clindamycin monotherapy remained significantly associated with favourable outcomes.

Another factor potentially contributing to the high failure rate in the combination group was the relatively short treatment duration. In our cohort, the median treatment length was 42 days, regardless of the presence of a foreign device. The DATIPO Trial demonstrated a higher failure rate in PJIs treated for 6 weeks compared with 12 weeks, with a difference in the risk of persistent infection (6 week group versus 12 week group) of 8.7 percentage points (95% CI, 1.8–15.6).^24^

Our results align with earlier studies supporting the efficacy of clindamycin monotherapy. Pontifex and McNaught^20^ first described monotherapy of clindamycin in BJI with a case series of 12 patients with only 2 treatment failures, 5 successes with sequelae and 5 complete successes. These patients were treated with doses lower than those used nowadays: 150 mg four times a day versus 600 mg three or four times a day in our study. Later, these data were confirmed in a rabbit model of osteitis caused by *S. aureus*^25^ and treated with clindamycin in monotherapy. The cure rate of this model was 84%. Courjon *et al.*^14^ published a cohort study on clindamycin-based treatment for BJI. In this cohort of 133 patients, only 15 patients received clindamycin in monotherapy, and no significant differences were found for treatment failure. *In vitro* studies have demonstrated clindamycin’s potent intracellular bactericidal activity against *S. aureus*, with a reduction of −5.2 log_10_ cfu/10^5^cells, and hence it could constitute an effective treatment for BJI on its own.^13^

More recently, a study comparing rifampicin-based therapy with a short-term (5 day) rifampicin regimen followed by clindamycin monotherapy for PJI managed with DAIR demonstrated comparable or even superior efficacy for the clindamycin-based regimen, achieving a 91% cure rate.^26^ The role of rifampicin in these infections has been increasingly debated: whereas some studies support its use in combination therapy, particularly in *S. aureus* PJI treated with DAIR, recent meta-analyses have failed to confirm its benefit across all pathogens^27^ and report only a marginal improvement in success rates for *S. aureus* PJI.^28^ These findings are consistent with our subgroup analysis, which supports the efficacy of clindamycin monotherapy in DAIR-managed PJI.

The similar efficiency or even superiority of monotherapy could be explained by fewer adverse events and most importantly by fewer drug–drug interactions. As in our study, a study looking at the occurrence of adverse events during treatment of BJI described only a few adverse events under clindamycin and macrolide group treatment.^29^ Drug–drug interaction with clindamycin is well documented, especially with rifampicin in combination. Induction of CYP3A4 by rifampicin is responsible for a major increase in clindamycin’s clearance and reduction of its exposure in BJI.^30^ The clinical impact of this interaction is not clearly identified but could result in clinical failure, which was not confirmed in our study. Recent pharmacokinetic data suggest that IV administration of clindamycin may mitigate the reduction in drug exposure caused by rifampicin-induced CYP3A4 activation, potentially preserving therapeutic levels.^31^ Although this route could represent a viable alternative when no other antibiotic options are suitable, clinical outcome data are still lacking and are needed to confirm the efficacy of this strategy.

### Conclusions

Clindamycin monotherapy appears to be a safe and effective treatment alternative for less severe BJI (CRP < 100 mg/L, absence of fever) in patients with fewer comorbidities (e.g. lower Charlson score, no chronic liver disease). These findings support the use of clindamycin as a standalone therapy in appropriate clinical contexts and highlight the need for prospective trials to confirm its efficacy and safety.

## Supplementary Material

dlaf164_Supplementary_Data

## Supplementary data

Figures S1 and S2 and Tables S1 to S4 are available as Supplementary data at JAC-AMR Online.

## References

[1] Grammatico-Guillon L, Baron S, Gettner S et al Bone and joint infections in hospitalized patients in France, 2008: clinical and economic outcomes. J Hosp Infect 2012; 82: 40–8. 10.1016/j.jhin.2012.04.025 22738613

[2] Laurent E, Gras G, Druon J et al Key features of bone and joint infections following the implementation of reference centers in France. Med Mal Infect 2018; 48: 256–62. 10.1016/j.medmal.2018.02.004 29526340

[3] Kehrer M, Pedersen C, Jensen TG et al Increasing incidence of pyogenic spondylodiscitis: a 14-year population-based study. J Infect 2014; 68: 313–20. 10.1016/j.jinf.2013.11.011 24296494

[4] Zimmerli W, ed . Bone and Joint Infections. Wiley, 2021.

[5] Ferry T, Seng P, Mainard D et al The CRIOAc healthcare network in France: a nationwide Health Ministry program to improve the management of bone and joint infection. Orthop Traumatol Surg Res 2019; 105: 185–90. 10.1016/j.otsr.2018.09.016 30413338

[6] Lemaignen A, Bernard L, Marmor S et al Epidemiology of complex bone and joint infections in France using a national registry: the CRIOAc network. J Infect 2021; 82: 199–206. 10.1016/j.jinf.2020.12.010 33352213

[7] Société de Pathologie Infectieuse de Langue Française (SPILF) . Recommandations de pratique clinique : infections ostéo-articulaires sur matériel (prothèse, implant, ostéosynthèse). 2009. https://www.infectiologie.com/UserFiles/File/spilf/recos/inf-osseuse-court.pdf 10.1016/j.medmal.2009.05.003 19877362

[8] Lacasse M, Derolez S, Bonnet E et al 2022 SPILF - Clinical Practice guidelines for the diagnosis and treatment of disco-vertebral infection in adults. Infectious Disease Now 2023; 53: 104647. 10.1016/j.idnow.2023.01.007 36690329

[9] Haute Autorité de Santé (HAS) . Recommandation de bonne pratique : prothèse de hanche ou de genou : diagnostic et prise en charge de l’infection dans le mois suivant l’implantation. 2014. www.has-sante.fr

[10] Leigh DA . Antibacterial activity and pharmacokinetics of clindamycin. J Antimicrob Chemother 1981; 7: 3–9. 10.1093/jac/7.suppl_A.3 7019193

[11] Schurman DJ, Johnson BL, Finerman G et al Antibiotic bone penetration. Concentrations of methicillin and clindamycin phosphate in human bone taken during total hip replacement. Clin Orthop Relat Res 1975; 111: 142–6. 10.1097/00003086-197509000-00019 1157411

[12] Thabit AK, Fatani DF, Bamakhrama MS et al Antibiotic penetration into bone and joints: an updated review. Int J Infect Dis 2019; 81: 128–36. 10.1016/j.ijid.2019.02.005 30772469

[13] Valour F, Trouillet-Assant S, Riffard N et al Antimicrobial activity against intraosteoblastic *Staphylococcus aureus*. Antimicrob Agents Chemother 2015; 59: 2029–36. 10.1128/AAC.04359-14 25605365 PMC4356812

[14] Courjon J, Demonchy E, Cua E et al Efficacy and safety of clindamycin-based treatment for bone and joint infections: a cohort study. Eur J Clin Microbiol Infect Dis 2017; 36: 2513–8. 10.1007/s10096-017-3094-5 28884303

[15] Smieja M . Current indications for the use of clindamycin: a critical review. Can J Infect Dis 1998; 9: 22–8. 10.1155/1998/538090 22346533 PMC3250868

[16] El Samad Y, Havet E, Bentayeb H et al Traitement des infections ostéoarticulaires par clindamycine chez l’adulte. Med Mal Infect 2008; 38: 465–70. 10.1016/j.medmal.2008.06.030 18718729

[17] Bernard A, Kermarrec G, Parize P et al Dramatic reduction of clindamycin serum concentration in staphylococcal osteoarticular infection patients treated with the oral clindamycin-rifampicin combination. J Infect 2015; 71: 200–6. 10.1016/j.jinf.2015.03.013 25936632

[18] Mimram L, Magréault S, Kerroumi Y et al What clindamycin dose should be administered by continuous infusion during combination therapy with rifampicin? A prospective population pharmacokinetics study. J Antimicrob Chemother 2023; 78: 2943–9. 10.1093/jac/dkad335 37883695

[19] Czekaj J, Dinh A, Moldovan A et al Efficacy of a combined oral clindamycin–rifampicin regimen for therapy of staphylococcal osteoarticular infections. Scand J Infect Dis 2011; 43: 962–7. 10.3109/00365548.2011.608082 21916775

[20] Pontifex AH, McNaught DR. The treatment of chronic osteomyelitis with clindamycin. Can Med Assoc J 1973; 109: 105–7. 4578855 PMC1946792

[21] Heinze G, Dunkler D. Five myths about variable selection. Transpl Int 2017; 30: 6–10. 10.1111/tri.12895 27896874

[22] Dziak JJ, Coffman DL, Lanza ST et al Sensitivity and specificity of information criteria. Brief Bioinform 2020; 21: 553–65. 10.1093/bib/bbz016 30895308 PMC7299313

[23] Austin PC, Stuart EA. Moving towards best practice when using inverse probability of treatment weighting (IPTW) using the propensity score to estimate causal treatment effects in observational studies. Stat Med 2015; 34: 3661–79. 10.1002/sim.6607 26238958 PMC4626409

[24] Bernard L, Arvieux C, Brunschweiler B et al Antibiotic therapy for 6 or 12 weeks for prosthetic joint infection. N Engl J Med 2021; 384: 1991–2001. 10.1056/NEJMoa2020198 34042388

[25] Norden CW, Shinners E, Niederriter K. Clindamycin treatment of experimental chronic osteomyelitis due to *Staphylococcus aureus*. J Infect Dis 1986; 153: 956–9. 10.1093/infdis/153.5.956 3701108

[26] Scheper H, van der Wal RJP, Mahdad R et al Effectiveness of different antimicrobial strategies for staphylococcal prosthetic joint infection: results from a large prospective registry-based cohort study. Open Forum Infect Dis 2022; 9: ofac474. 10.1093/ofid/ofac474 36225743 PMC9547512

[27] Aydın O, Ergen P, Ozturan B et al Rifampin-accompanied antibiotic regimens in the treatment of prosthetic joint infections: a frequentist and Bayesian meta-analysis of current evidence. Eur J Clin Microbiol Infect Dis 2021; 40: 665–71. 10.1007/s10096-020-04083-4 33125602

[28] Scheper H, Gerritsen LM, Pijls BG et al Outcome of debridement, antibiotics, and implant retention for staphylococcal hip and knee prosthetic joint infections, focused on rifampicin use: a systematic review and meta-analysis. Open Forum Infect Dis 2021; 8: ofab298. 10.1093/ofid/ofab298 34258321 PMC8271145

[29] Valour F, Karsenty J, Bouaziz A et al Antimicrobial-related severe adverse events during treatment of bone and joint infection due to methicillin-susceptible *Staphylococcus aureus*. Antimicrob Agents Chemother 2014; 58: 746–55. 10.1128/AAC.02032-13 24247130 PMC3910824

[30] Goulenok T, Seurat J, Selle AdL et al Pharmacokinetic interaction between rifampicin and clindamycin in staphylococcal osteoarticular infections. Int J Antimicrob Agents 2023; 62: 106885. 10.1016/j.ijantimicag.2023.106885 37302771

[31] Maugreault S, Berrah R, Kerroumi Y et al Dosing and route of administration of clindamycin given in combination with rifampicin. Clin Microbiol Infect 2025; 31: 832–8. 10.1016/j.cmi.2025.01.005 39827992

