# Supplementary material for: Evaluation of clindamycin use in bone and joint infections: is monotherapy a safe option? A monocentric observational study 2014–19
Source: JAC Antimicrob Resist. 2025 Sep 23;7(5):dlaf164. doi: 10.1093/jacamr/dlaf164 (PMC12455409; doi:10.1093/jacamr/dlaf164)
Supplement: dlaf164_Supplementary_Data [file dlaf164_supplementary_data.docx]

**Supplementary data**

**Table S1: Variable considered in propensity score model**

| Age (Continuous) |
| --- |
| Sex (Male/Female) |
| Ischemic heart disease (Y/N) |
| Chronic heart failure (Y/N) |
| Arterial occlusive diseases (Y/N) |
| Stroke (Y/N) |
| Dementia (Y/N) |
| Hemiplegia/Paraplegia/Quadriplegia (Y/N) |
| Diabetes mellitus (Y/N) |
| Gastro-intestinal ulcer (Y/N) |
| Cirrhosis stage B or more (Y/N) |
| Chronic kidney failure, eGFR <60mL/min (Y/N) |
| Chronic Respiratory pathology (Y/N) |
| Inflammatory rheumatism (Y/N) |
| Haematological malignancy (Yes/No) |
| Malignant Neoplasm (Yes/No) |
| Uncontrolled HIV infection or HIV infection with CD4 cell counts < 200/µl (Y/N) |
| Active smoking habit (Y/N) |
| BMI > 30 kg/m² (Y/N) |
| First episode of Bone and Joint Infection (Y/N) |
| Infection related to a surgical device (Y/N) |
| Polymicrobial infection (Y/N) |
| Infection due to *Staphylococcus aureus* (Y/N) |
| Fever at the time of diagnosis (Y/N) |
| CRP level at the time of diagnosis (Continuous) |

**Table S2: comparison of population characteristics before and after Inverse Propensity Score Weighting**

|  | **Before IPTW** | |  | **After IPTW** | |  |
| --- | --- | --- | --- | --- | --- | --- |
|  | **In combination** | **Monotherapy** | ***p-value*** | **In combination** | **Monotherapy** | ***p-value*** |
| **Demographics** |  |  |  |  |  |  |
| Sex *(Male)* | 73.5% | 71.6% | 0.97 | 68% | 75.9% | 0.32 |
| Age *(years)* *Median (IQR)* | 66.00 (22.00) | 65.50 (21.25) | 0.36 | 47.06(32.65) | 54.11 (28.86) | 0.22 |
|  |  |  |  |  |  |  |
| **Medical history** |  |  |  |  |  |  |
| Charlson score *Median (IQR)* | 6.00 (6.00) | 5.00 (5.00) | 0.08 | 3.95 (4.82) | 4.42 (4.80) | 0.65 |
| Chronic kidney failure, eGFR<60mL/min | 28.6% | 23.9% | 0.68 | 21.8% | 23% | >0.99 |
| Chronic heart failure | 24.5% | 31.8% | 0.48 | 24.5% | 36% | 0.18 |
| Ischemic heart disease | 12.2% | 12.5% | >0.99 | 11.9% | 21.2% | 0.25 |
| Arterial occlusive diseases | 12.2% | 18.2% | 0.51 | 11% | 15.3% | 0.6 |
| Stroke | 12.2% | 10.2% | 0.94 | 8.6% | 8.6% | >0.99 |
| Hemiplegia/Paraplegia/Quadriplegia | 6.1% | 2.3% | 0.49 | 4.5% | 2.5% | >0.99 |
| Dementia | 10.2% | 5.7% | 0.53 | 8.3% | 7.4% | >0.99 |
| Obesity (BMI>30kg/m²) | 30.6% | 27.3% | 0.83 | 28.6% | 36.1% | 0.45 |
| Malnutrition | 4.1% | 5.7% | 0.99 | 2.5% | 4.7% | 0.65 |
| Chronic Respiratory pathology | 26.5% | 15.9% | 0.20 | 22.3% | 18.4% | 0.65 |
| Cirrhosis stage B or more | 18.4% | 4.5% | **0.02*** | 9.3% | 14.8% | 0.6 |
| Diabetes mellitus | 46.9% | 46.6% | >0.99 | 36.9% | 43.4% | 0.48 |
| Malignant Neoplasm | 18.4% | 21.6% | 0.82 | 18.6% | 18.8% | >0.99 |
| Haematological malignancy | 2% | 3.4% | >0.99 | 1.6% | 2.7% | >0.99 |
| Immunodeficiency | 20.4% | 19.3% | >0.99 | 12.3% | 15.8% | 0.63 |
| Uncontrolled HIV infection or HIV infection with CD4 cell counts < 200/µl | 4.1% | 1.1% | 0.60 | 2.5% | 1.9% | >0.99 |
| Inflammatory rheumatism | 10.2% | 12.5% | 0.90 | 6.7% | 10% | 0.54 |
| Active smoking habit | 51% | 36.4% | 0.14 | 45.5% | 50% | 0.6 |
| Chronic alcoholic intoxication | 46.9% | 28.4% | **0.04*** | 42.1% | 26.3% | 0.06 |
|  |  |  |  |  |  |  |
| **Characteristics of infection** |  |  |  |  |  |  |
| Device related infection | 55.1% | 64.8% | 0,35 | 62.1% | 69.2% | 0.35 |
| Chronic infection | 77.6% | 71.6% | 0.58 | 82.7% | 74.3% | 0.21 |
| First episode of infection | 57.1% | 61.4% | 0.76 | 62.7% | 57.3% | 0.48 |
| C-Reactive Protein *(mg/L) Median (IQR)* | 102.6 (104) | 65.7 (80.8) | **0.006*** | 62.13 (80.7) | 50.87(84.67) | 0.06 |
| Fever | 51% | 32.9% | 0.06 | 37.7% | 45.9% | 0.37 |
|  |  |  |  |  |  |  |
| **Microbiological identification** |  |  |  |  |  |  |
| Polymicrobial infection | 24.5% | 30.7% | 0.57 | 33.9% | 36.9% | >0.99 |
| *Staphylococcus aureus* | 59.2% | 59.1% | >0.99 | 57.9% | 65.8% | 0.36 |
| Coagulase negative *Staphylococci* | 32.6% | 27.3% | 0.64 | 31.8% | 25.1% | 0.43 |
| *Cutibacterium sp* | 10.2% | 17.1% | 0.40 | ¨18.3% | 13.9% | 0.47 |
|  |  |  |  |  |  |  |
| **Treatment and outcome** |  |  |  |  |  |  |
| Surgery | 91.8% | 93.2% | >0.99 | 90.4% | 97.7% | 0.1 |
| Duration of treatment *(day) Median (IQR)* | 42 (3.00) | 42 (3) | 0.25 | 33.69 (28.27) | 36.39 (32.27) | 0.26 |
| Failure | 51% | 18.2% | **<0.001*** | 45.7% | 25.9% | **0.03*** |

Unless otherwise indicated, data are presented as a percentage distribution of patients

**Table S3: Complete logistic regression analysis for treatment outcomes**

|  | **Success (n=96)** | **Failure  (n=41)** | **Univariate OR (IC95, p)** | **Multivariate**  **OR (IC95, p)** | **Multivariate adjusted on AIC OR (IC95, p)** | **IPTW**  **OR (IC95, p)** |
| --- | --- | --- | --- | --- | --- | --- |
| Sex *(Male)* | 71 (74.0) | 28 (68.3) | 0.76 (0.34-1.72, p=0.5) | - | - | - |
| Age *(years)* *Median (IQR)* | 63.50 (22.25) | 70.00 (19.00) | 1.01 (0.99-1.04, p=0.2) | 1.01 (0.97-1.04, p=0.71) | - | - |
| Chronic kidney failure, eGFR<60mL/min | 24 (25.0) | 11 (26.8) | 1.10 (0.47-2.49, p=0.82) | - | - | - |
| Chronic heart failure | 26 (27.1) | 14 (34.1) | 1.40 (0.63-3.05, p=0.41) | - | - | - |
| Ischemic heart disease | 9 (9.4) | 8 (19.5) | 2.34 (0.82-6.64, p=0.11) | 2.85 (0.72-11.69, p=0.13) | 3.17 (0.89-11.66, p=0.07) | - |
| Arterial occlusive diseases | 11 (11.5) | 11 (26.8) | 2.83 (1.11-7.29, p=0.03) | 1.91 (0.46-7.87, p=0.34) | 2.81 (0.89-9.07, p=0.08) | - |
| Stroke | 10 (10.4) | 5 (12.2) | 1.19 (0.35-3.62, p=0.76) | - | - | - |
| Dementia | 6 (6.2) | 4 (9.8) | 1.62 (0.40-6.01, p=0.47) | - | - | - |
| Obesity (BMI>30kg/m²) | 23 (24.0) | 16 (39.0) | 2.03 (0.92-4.45, p=0.08) | 1.65 (0.55-4.78, p=0.36) | - | - |
| Malnutrition | 2 (2.1) | 5 (12.2) | 6.53 (1.34-47.03, p=0.03) | 11.83 (1.61-118.87, p=0.02) | 11.83 (1.74-112.31, p=0.02) | - |
| Chronic Respiratory pathology | 14 (14.6) | 13 (31.7) | 2.72 (1.13-6.53, p=0.02) | 1.35 (0.40-4.34, p=0.61) | - | - |
| Cirrhosis stage B or more | 5 (5.2) | 8 (19.5) | 4.41 (1.37-15.51, p=0.01) | 2.05 (0.39-11.73, p=0.4) | 3.17 (0.80-13.64, p=0.1) | - |
| Diabetes mellitus | 41 (42.7) | 23 (56.1) | 1.71 (0.82-3.62, p=0.15) | 1.20 (0.45-3.22, p=0.71) | - | - |
| Malignant Neoplasm | 17 (17.7) | 11 (26.8) | 1.70 (0.70-4.03, p=0.23) | 2.60 (0.80-8.70, p=0.11) | 2.43 (0.80-7.47, p=0.12) | - |
| Immunodeficiency | 21 (21.9) | 6 (14.6) | 0.61 (0.21-1.57, p=0.33) | - | - | - |
| Uncontrolled HIV infection or HIV infection with CD4 cell counts < 200/µl | 1 (1.0) | 2 (4.9) | 4.87 (0.45-106.62, p=0.2) | 2.97 (0.06-251.42, p=0.62) | - | - |
| Inflammatory rheumatism | 10 (10.4) | 6 (14.6) | 1.47 (0.47-4.29, p=0.48) | - | - | - |
| Active smoking habit | 38 (39.6) | 19 (46.3) | 1.32 (0.63-2.76, p=0.463) | - | - | - |
| Chronic alcoholic intoxication | 29 (30.2) | 19 (46.3) | 2.00 (0.94-4.25, p=0.07) | 1.46 (0.52-4.10, p=0.46) | - | - |
| Site of infection |  |  |  |  |  |  |
| Lower limbs | 66 (68.8) | 29 (70.7) | 0.66 (0.10-5.20, p=0.66) | - | - | - |
| Upper limbs | 27 (28.1) | 8 (19.5) | 0.44 (0.06-3.81, p=0.42) | - | - | - |
| Arthritis | 16 (16.7) | 6 (14.6) | 0.86 (0.29-2.28, p=0.77) | - | - | - |
| Osteitis | 22 (22.9) | 8 (19.5) | 0.82 (0.31-1.96, p=0.66) | - | - | - |
| Diabetic foot related infection | 6 (6.2) | 3 (7.3) | 1.18 (0.24-4.74, p=0.82) | - | - | - |
| Device related infection | 59 (61.5) | 25 (61.0) | 0.98 (0.47-2.10, p=0.96) | - | - | - |
| Of which: Prosthetic Joint Infection | 32 (33.3) | 13 (31.7) | 0.93 (0.42-2.01, p=0.85) | - | - | - |
| Type of prothesis |  |  |  |  |  |  |
| Knee | 3 (3.1) | 5 (12.2) | 3.94 (0.91-20.23, p=0.07) | 3.93 (0.52-34.28, p=0.19) | - | - |
| Hip | 22 (22.9) | 6 (14.6) | 0.65 (0.22-1.67, p=0.39) | - | - | - |
| Chronic infection | 71 (74.0) | 30 (73.2) | 0.96 (0.43-2.26, p=0.92) | - | - | - |
| First episode of infection | 57 (59.4) | 25 (61.0) | 1.07 (0.51-2.29, p=0.86) | - | - | - |
| C-Reactive Protein *(mg/L) Median (IQR)* | 64.85 (82.85) | 102.6 (124.0) | 1.01 (1.00-1.01, p=0.008) | 1.00 (0.99-1.01, p=0.87) | - | - |
| Fever | 30 (31.2) | 24 (58.5) | 3.11 (1.47-6.71, p=0.003) | 2.88 (1.00-8.73, p=0.05) | 3.26 (1.30-8.56, p=0.01) | - |
| Polymicrobial infection | 29 (30.2) | 10 (24.4) | 0.75 (0.31-1.68, p=0.49) | - | - | - |
| *Staphylococcus aureus* | 54 (56.2) | 27 (65.9) | 1.50 (0.71-3.27, p=0.3) | 1.03 (0.36-2.98, p=0.96) | - | - |
| Coagulase negative *Staphylococci* | 28 (29.2) | 12 (29.3) | 1.00 (0.44-2.22, p=0.99) | - | - | - |
| *Cutibacterium sp* | 16 (16.7) | 4 (9.8) | 0.54 (0.15-1.60, p=0.3) | 0.84 (0.15-4.04, p=0.84) | - | - |
| Surgery | 90 (93.8) | 37 (90.2) | 0.62 (0.17-2.53, p=0.47) | - | - | - |
| Monotherapy | 72 (75.0) | 16 (39.0) | 0.21 (0.10-0.46, p<0.001) | 0.19 (0.06-0.51, p=0.001) | 0.18 (0.07-0.46, p<0.001) | 0.36 (0.17-0.76, p=0.008) |
| Duration of treatment *(days) Median (IQR)* | 42.0 (3.0) | 42.0 (3.0) | 1.00 (0.99-1.02, p=0.57) | - | - | - |

**Table S4: Complete univariate analysis of risk factors for Adverse events**

|  | **No Adverse event (n=125)** | **Adverse event  (n=12)** | **Univariate OR (IC95, p)** |
| --- | --- | --- | --- |
| Sex *(Male)* | 91 (72.8) | 8 (66.7) | 0.75 (0.22-2.95, p=0.65) |
| Age *(years)* *Median (IQR)* | 64 (21) | 73 (23) | 1.04 (1.00-1.10, p=0.05) |
| Chronic kidney failure, eGFR<60mL/min | 30 (24.0) | 5 (41.7) | 2.26 (0.63-7.62, p=0.19) |
| Chronic heart failure | 37 (29.6) | 3 (25.0) | 0.79 (0.17-2.83, p=0.74) |
| Arterial occlusive diseases | 19 (15.2) | 3 (25.0) | 1.86 (0.39-6.91, p=0.38) |
| Stroke | 13 (10.4) | 2 (16.7) | 1.72 (0.25-7.50, p=0.51) |
| Dementia | 9 (7.2) | 1 (8.3) | 1.17 (0.06-7.15, p=0.88) |
| Obesity (BMI>30kg/m²) | 36 (28.8) | 3 (25.0) | 0.82 (0.18-2.95, p=0.78) |
| Chronic Respiratory pathology | 21 (16.8) | 6 (50.0) | 4.95 (1.42-17.33, p=0.01) |
| Cirrhosis stage B or more | 12 (9.6) | 1 (8.3) | 0.86 (0.04-5.02, p=0.89) |
| Diabetes mellitus | 57 (45.6) | 7 (58.3) | 1.67 (0.51-5.91, p=0.4) |
| Malignant Neoplasm | 26 (20.8) | 2 (16.7) | 0.76 (0.11-3.12, p=0.73) |
| Immunodeficiency | 24 (19.2) | 3 (25.0) | 1.40 (0.29-5.12, p=0.63) |
| Inflammatory rheumatism | 13 (10.4) | 3 (25.0) | 2.87 (0.58-11.12, p=0.14) |
| Active smoking habit | 51 (40.8) | 6 (50.0) | 1.45 (0.43-4.88, p=0.54) |
| Chronic alcoholic intoxication | 44 (35.2) | 4 (33.3) | 0.92 (0.23-3.10, p=0.9) |
| Arthritis | 21 (16.8) | 1 (8.3) | 0.45 (0.02-2.51, p=0.46) |
| Osteitis | 28 (22.4) | 2 (16.7) | 0.69 (0.10-2.83, p=0.65) |
| Device related infection | 74 (59.2) | 10 (83.3) | 3.45 (0.86-23.03, p=0.12) |
| Of which: Prosthetic Joint Infection | 40 (32.0) | 5 (41.7) | 1.52 (0.43-5.05, p=0.5) |
| Type of prothesis |  |  |  |
| Knee | 7 (5.6) | 1 (8.3) | 1.66 (0.08-11.16, p=0.65) |
| Hip | 25 (20.0) | 3 (25.0) | 1.39 (0.29-5.23, p=0.64) |
| Chronic infection | 93 (74.4) | 8 (66.7) | 0.69 (0.20-2.72, p=0.56) |
| First episode of infection | 73 (58.4) | 9 (75.0) | 2.14 (0.60-9.98, p=0.27) |
| C-Reactive Protein *(mg/L) Median (IQR)* | 68.1 (88.0) | 122.0(123.25) | 1.00 (1.00-1.01, p=0.15) |
| Fever | 46 (36.8) | 8 (66.7) | 3.43 (1.02-13.45, p=0.05) |
| Polymicrobial infection | 38 (30.4) | 1 (8.3) | 0.21 (0.01-1.13, p=0.14) |
| *Staphylococcus aureus* | 74 (59.2) | 7 (58.3) | 0.96 (0.29-3.42, p=0.95) |
| Coagulase negative *Staphylococci* | 36 (28.8) | 4 (33.3) | 1.24 (0.31-4.19, p=0.74) |
| *Cutibacterium sp* | 19 (15.2) | 1 (8.3) | 0.51 (0.03-2.85, p=0.53) |
| Surgery | 116 (92.8) | 11 (91.7) | 0.85 (0.14-16.47, p=0.88) |
| Monotherapy | 84 (67.2) | 4 (33.3) | 0.24 (0.06-0.82, p=0.03) |
| Duration of treatment *(days) Median (IQR)* | 42 (3) | 42 (0.75) | 1.00 (0.97-1.02, p=0.73) |

**Figure S1: Flow-Chart**

**Figure S2: Subgroup analysis of antibiotic combination**

**
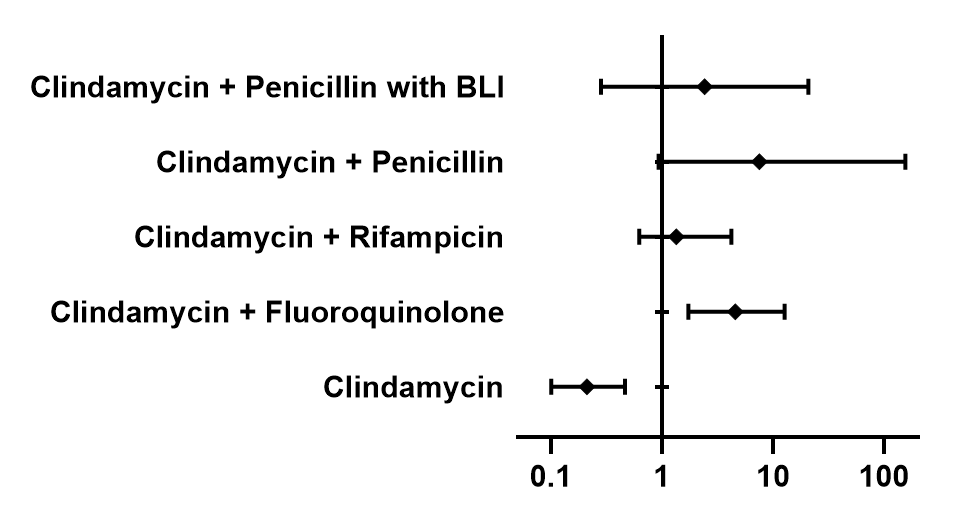
**Univariate logistic regression of subgroup analysis for main antibiotic combination (n>3) on failure. BLI = Beta-lactamase inhibitor

**Treatment** **Failure** **univariate**

Clindamycin + Penicillin with BLI 2/4 (50%) 2.41 (0.28-20.67, p=0.387)

Clindamycin + Penicillin 3/4 (75%) 7.50 (0.93-154.23, p=0.085)

Clindamycin + Rifampicin 5/14 (36%) 1.34 (0.39-4.17, p=0.619)

Clindamycin + Fluoroquinolone 12/20 (60%) 4.55 (1.72-12.68, p=0.003)

Clindamycin monotherapy 16/88 (18%) 0.21 (0.10-0.46, p<0.001)

Success Failure
